# Etiological factors in primary hepatic B-cell lymphoma

**DOI:** 10.1007/s00428-012-1199-x

**Published:** 2012-03-07

**Authors:** Kanta Kikuma, Jiro Watanabe, Yumi Oshiro, Tatsuo Shimogama, Yumi Honda, Seiichi Okamura, Koichi Higaki, Naokuni Uike, Tetsuro Soda, Seiya Momosaki, Tadaaki Yokota, Satoshi Toyoshima, Morishige Takeshita

**Affiliations:** 1 Departments of Pathology and Internal Medicine, Faculty of Medicine, Fukuoka University, Nanakuma 7-45-1, Johnan-ku, Fukuoka, Japan; 2 Pathological Laboratory, National Organization Kokura Hospital, Harugaoko 10-1, Kokura minmi-ku, Kitakyushu, Japan; 3 Pathological Laboratory, Matsuyama Red-Cross Hospital, Bunkyo-cho, 1-1, Matsuyama, Japan; 4 Pathological Laboratory, Nihon Steel Yawata Memorial Hospital, Horunomachi 1-1-1, Yawata nishi-ku, Kitakyushu, Japan; 5 Pathological Laboratory, Kumamoto University Hospital, Honjyo 1-1-1, Kumamoto, Japan; 6 Hematology Laboratory and Pathological Laboratory, National Organization Kyushu Medical Center, Jigyohama 1-8-1, Chuo-ku, Fukuoka, Japan; 7 Pathological Laboratory, Saint Mary Hospital, Tsufukumotomachi 422, Kurume, Japan; 8 Hematology Laboratory, National Organization Kyushu Cancer Center, Notamo 3-1-1, Minami-ku, Fukuoka, Japan; 9 Pathological Laboratory, Kokura Memorial Hospital, Kisencho 1-1, Kokura kita-ku, Kitakyushu, Japan; 10 Pathological Laboratory, Municipal Kitakyushu Medical Center, Bashaku 2-1-1, Kokura kita-ku, Kitakyushu, Japan

**Keywords:** Liver, Malignant lymphoma, HCV, Autoimmune disease

## Abstract

Sixty-four cases of malignant lymphoma involving the liver were examined. Of these, 20 cases were histologically confirmed to be primary hepatic B-cell lymphoma. Twelve of these 20 cases were diffuse large B-cell lymphoma (DLBCL) and eight cases were mucosa-associated lymphoid tissue (MALT) lymphoma. Of the 12 cases of DLBCL, six were immunohistologically positive for CD10 and/or Bcl6 (indicating a germinal center phenotype), six were positive for Bcl2, and five were positive for CD25. Eight of the 12 DLBCL cases (66.7%) and two of the eight MALT lymphoma cases (25%) had serum anti-hepatitis C virus (HCV) antibodies and HCV RNA. The incidence of HCV infection was significantly higher in the hepatic DLBCL cases than in systemic intravascular large B-cell cases with liver involvement (one of 11 cases, 9.1%) and T/NK-cell lymphoma cases (one of 19 cases, 5.3%) (*p* < 0.01 for both). Two hepatic DLBCL cases (16.7%) had rheumatoid arthritis treated with methotrexate, and four MALT lymphoma cases (50%) had Sjögren’s syndrome, primary biliary cirrhosis, or autoimmune hepatitis; one case in each of these two groups was complicated by chronic HCV-seropositive hepatitis. Although primary hepatic lymphoma is rare, persistent inflammatory processes associated with HCV infection or autoimmune disease may play independent roles in the lymphomagenesis of hepatic B cells.

## Introduction

B-cell lymphoma in hepatitis C virus (HCV)-seropositive patients frequently presents with extranodal disease in the bone marrow, major salivary glands, or spleen [1, 2]. Although diffuse large B-cell lymphoma (DLBCL) is frequent in HCV-seropositive B-cell lymphoma patients, a significantly higher incidence of lymphoplasmacytic lymphoma than HCV-negative B-cell lymphoma has been reported (43 of 257 cases, 16.7% versus 91 of 1118 cases, 8%; *p* < 0.01) [3]. Hermine et al. [4] described nine French HCV-seropositive cases of splenic marginal zone lymphoma with monoclonal IgM and polyclonal IgG (type II) cryoglobulinemia, and reported that all these cases showed loss of serum HCV RNA and complete remission of lymphoma after treatment with interferon-α and ribavirin. HCV infection and associated type II cryoglobulinemia may induce B-cell lymphoid neoplasia.

The HCV core protein has been shown to induce proliferation of human B-cell lines through up-regulation of specific B-cell-related genes and reduction in apoptosis [5, 6]. The levels of the activation-induced cytidine deaminase protein and gene, which provide a key enzyme for somatic hypermutation of germinal center (GC) B cells, were dramatically increased in peripheral blood B cells in patients with HCV-seropositive hepatitis [7]. HCV transgenic mice expressing the full HCV genome in B cells showed a high incidence of CD25 [interleukin-2α receptor (IL2αR)]-positive DLBCL as well as elevated serum levels of IL2αR [8]. HCV infection may play a role in the multistep mechanism of lymphomagenesis in peripheral B cells.

Ramos-Casals et al. [9] reported 25 cases of B-cell lymphoma with HCV infection and Sjögren’s syndrome (SS), which showed frequent type II cryoglobulinemia (80%), extranodal tumor invasion (60%) including in the liver (16%), mucosa-associated lymphoid tissue (MALT) lymphoma (44%), and DLBCL (24%). Primary biliary cirrhosis (PBC) has been reported to occasionally be complicated by HCV infection (14 of 170 cases, 8.2%), but not by lymphoma [10]. Autoimmune disease with or without HCV infection may play a role in primary hepatic lymphoma.

Primary hepatic B-cell lymphoma is rare, representing 0.06% of all non-Hodgkin’s lymphoma [11]. Page et al. [11] reported 23 cases of primary hepatic DLBCL and one of MALT lymphoma, of which six of the 10 examined DLBCL cases (60%) had HCV infection. Persistent HCV infection often complicates cases of primary hepatic DLBCL. We selected 20 cases of primary hepatic B-cell lymphoma from an area of Japan in which HCV infection is endemic. Of these, eight of 12 DLBCL cases (66.7%) and two of eight MALT lymphoma cases (25%) had serum anti-HCV antibodies and HCV RNA, and six DLBCL cases and four MALT lymphoma cases had autoimmune disease. We examined clinicopathological characteristics and etiological factors in patients with primary hepatic B-cell lymphoma, especially cases with HCV infection and autoimmune disease.

## Materials and methods

### Case selection

We reviewed the 5220 cases of malignant lymphoma in our register from 1995 to 2010, and included the 64 cases with histologically confirmed liver involvement at initial presentation in this study. Primary hepatic B-cell lymphoma was defined as extranodal lymphoma of the liver, with the bulk of the disease localized to this site [12]. Contiguous lymph node and perihepatic metastasis may be seen in primary hepatic B-cell lymphoma, as in gastrointestinal extranodal B-cell lymphoma [13, 14]. Histological diagnosis was according to the World Health Organization classification [13]. Twenty of 5,220 cases (0.4%) had clinical stage I or II primary hepatic lymphoma without superficial lymphadenopathy. The other 44 cases with liver involvement had malignant lymphoma with systemic or secondary intrahepatic tumor invasion as well as extrahepatic tumors or superficial nodal invasion. These 44 cases were used as a control group, comprising 22 cases of systemic B-cell lymphoma [12 cases of intravascular large B-cell lymphoma (IVLBCL) and 10 cases of DLBCL] and 22 cases of T/NK-cell lymphoma (nine cases of hepatosplenic T-cell lymphoma, seven cases of adult T-cell leukemia/lymphoma (ATL/L), four cases of aggressive NK-cell leukemia/lymphoma, and two cases of other T-cell lymphoma). In an additional control study, the seven cases of primary hepatic reactive lymphoid hyperplasia [14] were also examined to check for HCV infection and autoimmune disease.

The clinical and laboratory findings, treatment, and prognosis of patients presenting to ten hospitals were examined. Tumor stages were classified according to the modified Ann Arbor staging system [15].

### Examination for HCV and HBV infections

Serum samples were screened for HCV infection by measuring anti-HCV antibody levels using the second generation enzyme-linked immunosorbent assay (ELISA) technique (Abbott Laboratories, Chicago, IL, USA). If positive, a PCR-based technique (PCR Amplicor; Roche, Branchburg, NJ, USA) was used to detect HCV RNA. The HCV core genotype was determined using PCR-based techniques (SMI test HCV genotyping kit; Sumitomo Metals, Osaka, Japan), which identified six genotypes according to the Simmonds classification: 1A, 1B, 2A, 2B, 3A, and 3B. HBV surface (HBs) and envelope (HBe) antigens were detected using an ELISA kit (Abbott Laboratories, Abbott Park, IL, USA) [16].

### Histology and immunohistology

Biopsy and surgical specimens were fixed with 20% formalin, embedded in paraffin, and stained with hematoxylin and eosin. The hepatic lesions of all 71 cases were examined histologically, including 16 autopsy cases (two primary hepatic DLBCL cases, two IVLBCL cases, and 12 T/NK-cell lymphoma cases). Immunohistological examination was performed by applying a panel of monoclonal and polyclonal antibodies to formalin-fixed tumor samples using the ChemMate Envision method (DakoCytomation, Glostrup, Denmark), and the peroxidase color reaction was developed using diaminobenzidine as the substrate. Antibody, clone, and source were as follows: CD3 (PS1, Novocastra, UK), CD5 (4C7, Novocastra), CD10 (56C6, Novocastra), CD20 (L26, Dako, USA), CD23 (1B23, Novocastra), IL2α receptor (CD25, 4C9, Novocastra), CD30 (BerH2, Dako), Bcl1 (5D4, IBL, Japan), Bcl2 (124, Dako), Bcl6 (P1F6, Novocastra), multiple myeloma 1 (MUM1p, Dako), anti-HBs antigen (ZCH16, Nichirei, Japan), and IgG4 (HP6026, Zymed, USA).

### In situ hybridization for detection of Epstein–Barr virus-encoded RNA (EBER)

Deparaffinized tissue sections were digested with proteinase K and hybridized in a solution of 50% formamide containing fluorescein isothiocyanate (FITC)-labeled EBER oligonucleotides using a Dako hybridization kit (DakoCytomation, Glostrup, Denmark). Hybridization products were detected using rabbit anti-FITC antibody, then incubated with ChemMate Envision and colored with DAB.

### Statistical analyses

Univariate analyses were performed using the chi-square and Fisher’s tests to determine differences between the two malignant lymphoma groups. Fifty-four node-based DLBCL cases and 445 colon cancer cases admitted to the National Kyushu Medical Center were selected for examination of HCV and HBV infections as a control group. Prognosis was determined by calculating the cumulative survival time of 53 cases using the Kaplan–Meier method and was analyzed using the log-rank and generalized Wilcoxon tests.

### Selection of 36 previously reported primary hepatic B-cell lymphoma cases

Sixteen primary DLBCL cases and 20 MALT lymphoma cases, which had each been described in detail, were selected from 27 reports in the English literature from 1990 to 2010 [17–42]. The clinical and histological details of these 36 cases were determined from the reported findings by the corresponding laboratory.

## Results

### Clinical findings, laboratory data, and outcomes

The clinical data at initial presentation of the 20 cases of primary hepatic lymphoma, 44 cases of systemic B- and T/NK-cell lymphoma with liver involvement, and seven cases of reactive lymphoid hyperplasia are shown in Table 1. Twelve cases had primary hepatic DLBCL. Eight of these 12 cases (66.7%) had serum anti-HCV antibodies, and HCV RNA was detected in all six of these cases examined. Two of these cases were infected with genotype 1b and two with genotype 2a. The prevalence of anti-HCV antibody positivity was significantly higher in DLBCL cases (66.7%) than in cases of systemic IVLBCL (one of 11, 9.1%), systemic T/NK-cell lymphoma (one of 19, 5.3%), nodal DLBCL (five of 54, 9.3%), and colon cancer (23 of 445, 5.2%) (*p* < 0.01). Two other DLBCL cases (16.7%) had HBs and HBe antigens, but this prevalence was not significantly higher than in cases of systemic IVLBCL (one of 11, 9.1%), nodal DLBCL (one of 54, 1.9%), or colon cancer (seven of 445, 1.6%). Eight cases had primary hepatic MALT lymphoma. Two of these cases (25%) were positive for serum anti-HCV antibodies and HCV RNA, and were infected with genotype 1b. This HCV prevalence was not significantly higher than in the other four tumor groups described above. In the other two groups of patients with systemic B-cell lymphoma, five of the ten DLBCL cases (50%) had anti-HCV antibodies. One case (14.3%) with reactive lymphoid hyperplasia had HCV infection. There were no cases with co-infection of HCV and HBV.

**Table 1:** Initial clinical data of 71 cases of liver-involving lymphoproliferative disease

No. of cases	Age (mean)	M/F	Histological type: no. of cases	Anti-HCV Ab	HBs, e Ag	Anti-HTLV-1 Ab
Primary hepatic B-cell lymphoma						
20	64 years old	10:2	DLBCL: 12	8 (66.7%)	2 (16.7%)	1 (8.3%)
		3:5	MALT: 8	2 (25%)	1 (12.5%)	0/5 (0)
Total		13:7		10 (50%)	3 (15%)	1/17 (5.9%)
Liver-involving systemic B-cell lymphoma						
22	67 years old	7:5	IVLBCL: 12	1/11 (9.1%)	1/11 (9.1%)	1/10 (10%)
		5:5	DLBCL: 10	5 (50%)	1 (10%)	0/7 (0)
Total		12:10		6/21 (29%)	2/21 (9.5%)	1/17 (5.9%)
Liver-involving systematic T/NK-cell lymphoma						
22	55 years old	17:5		1/19 (5.3%)	0/19 (0)	7/21 (33.3%)
Reactive lymphoid hyperplasia						
7	61 years old	0:7		1(14.3%)	0 (0)	0 (0)

/: positive/examined cases. *HCV* hepatitis C virus, *HB* hepatitis B, *HTLV* human T-cell lymphotropic virus, *DLBCL* diffuse large B-cell lymphoma, *MALT* mucosa-associated lymphoid tissue, *IVLBCL* intravascular large B-cell lymphoma

Detailed clinicopathological findings of the primary hepatic and systemic B-cell lymphoma cases are shown in Table 2. Of the cases with primary hepatic DLBCL, five had chronic hepatitis, three had liver cirrhosis, and one had chronic hepatitis and hepatocellular carcinoma. The remaining case was an HCV carrier. Two cases had rheumatoid arthritis which had been treated with methotrexate for 7 and 10 years, respectively, and one case had chronic HCV hepatitis. Seven cases were classified as clinical stage I and five were classified as II1 or II2. Combined chemotherapy was administered in nine cases. The five cases who underwent rituximab plus cytotoxic treatment were alive and well at 21 to 72 months after treatment initiation, while three of the four cases who received only cytotoxic treatment died of disease with extrahepatic tumor invasion. The median survival time of these nine treated cases was 27 months, and the 5-year overall survival rate was 44%. Two cases did not receive cytotoxic treatment and died of hepatic failure. Limited intrahepatic lymphoma was found at autopsy. The remaining case was lost to follow-up after 3 months.

**Table 2:** Detailed clinicopathological findings and prognosis in 42 of liver-involving B-cell lymphoma

Histological type	Clinical stage	Liver disease			Hepatic tumors			Complication	Median survival (months)
	I/II/III/IV	CH	LC	HCC	Single	Multiple	Diffuse		
Primary hepatic B-cell lymphoma									
DLBCL: 12	7/5/0//0	5	3	1	8	4	0	RA: 2	27
MALT: 8	8/0/0//0	3	1	1	8	0	0	SS: 2, PBC: 1^a^, AH: 1	>84
Total: 20	15/5/0//0	8	4	2	16	4	0	6	
Liver-involving systemic B-cell lymphoma									
IVLBCL: 12	0/0/0//12	1	0	0	0	0	12	SLE: 1	6
DLBCL: 10	0/0/0//10	5	0	0	2	8	0	0	13
Total 22	0/0/0//22	6	0	0	2	8	12	1	

*DLBCL* diffuse large B-cell lymphoma, *MALT* mucosa-associated lymphoid tissue, *IVLBCL* intravascular large B-cell lymphoma, *CH* chronic hepatitis, *LC* liver cirrhosis, *HCC* hepatocellular carcinoma, *RA* rheumatoid arthritis, *SS* Sjögren’s syndrome, *PBC* primary biliary cirrhosis, *AH* autoimmune hepatitis, *SLE* systemic lupus erythematosus

^a^One patient complicates PBC and SS

**Fig. 1:**
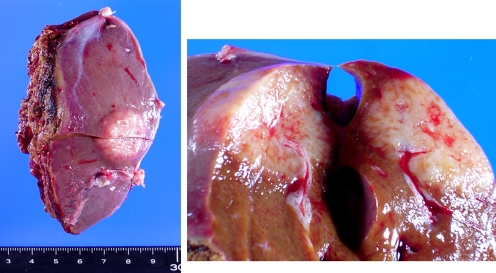
Macroscopic appearance of a primary hepatic MALT lymphoma without HCV infection. A whitish nodular tumor is seen in the liver

**Fig. 2:**
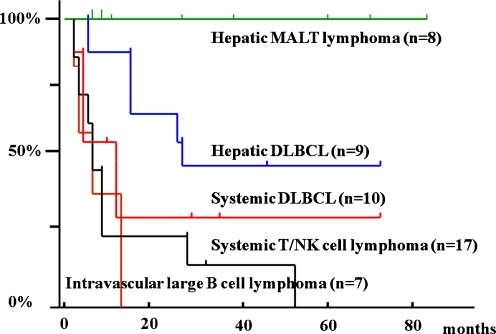
Actuarial survival curves of cases of primary hepatic DLBCL and MALT lymphoma, intravascular and systemic large B-cell lymphoma, and systemic T/NK-cell lymphoma with liver involvement. Primary hepatic MALT lymphoma cases have a significantly better prognosis than primary hepatic DLBCL cases (*p* < 0.05). Primary hepatic DLBCL cases have a significantly better prognosis than cases of intravascular large B-cell lymphoma, systemic DLBCL, and systemic T/NK-cell lymphoma (*p* < 0.01 or <0.05)

All eight cases of primary hepatic MALT lymphoma had a single hepatic tumor (Fig. 1). Two cases had SS, one had SS and PBC, and one had autoimmune hepatitis. All these cases received surgery and/or cytotoxic treatment with or without rituximab, and were alive and well at 5 to 84 months after treatment initiation. Their median survival time was longer than 84 months, which was significantly longer than that of primary hepatic DLBCL cases (*p* < 0.05) (Fig. 2).

In cases of systemic lymphoma with liver involvement, only one of 12 IVLBCL cases (8.3%) had systemic lupus erythematosus. The prevalence of autoimmune disease was significantly lower in IVLBCL cases than in primary hepatic lymphoma cases (six of 20 cases, 30%; *p* < 0.05). The median survival time of the seven IVLBCL cases for which this information was available was 6 months, which was significantly shorter than in primary hepatic DLBCL cases (*p* < 0.01). Of the ten cases with systemic DLBCL, four patients with anti-HCV antibodies died of disease within 6 months. The median survival time of these ten cases was 13 months, which was significantly shorter than in primary hepatic DLBCL cases (*p* < 0.05). Of the 22 cases of systemic T/NK-cell lymphoma, one case with T-LGL leukemia (5.3%) had PBC. One case with reactive lymphoid hyperplasia (14.3%) was complicated by PBC.

### Histological and immunohistological findings

**Fig. 3:**
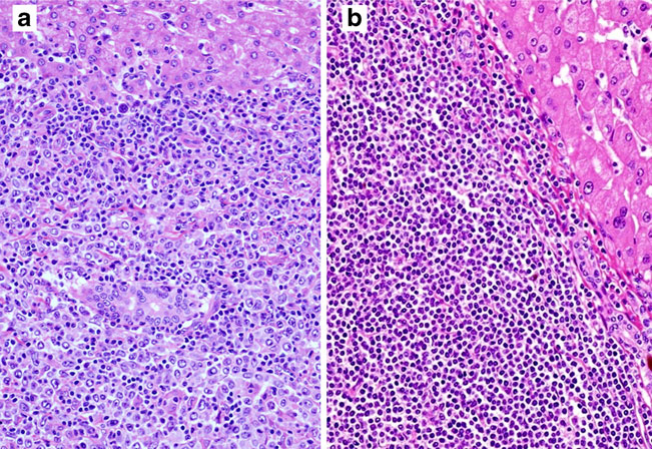
Histological appearance of primary hepatic DLBCL (**a**) and MALT lymphoma (**b**) in HCV-seropositive cases (hematoxylin and eosin staining, ×400). In the DLBCL case, many small lymphocytes are seen among large lymphoid cells

**Fig. 4:**
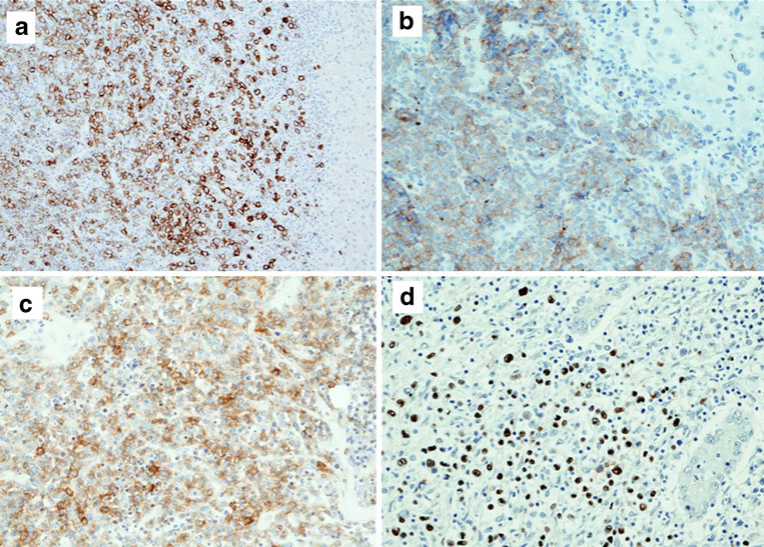
HCV-seropositive primary hepatic DLBCL. **a, b, c** Immunoperoxidase staining. **d** In situ hybridization, ×200. **a** Many CD20-positive large lymphoid cells show a diffuse infiltrating pattern. **b** CD10-positive large lymphoid cells are diffusely distributed. Bile canaliculi have focal weak CD10-positivity in the upper right hepatic lobule. **c** Large lymphoid cells are positive for CD25. **d** Many nuclear EBER signals are detected in medium-sized and large lymphoid cells. Two bile ducts on the right are preserved

The immunohistological characteristics of the primary hepatic and systemic B-cell lymphoma cases are shown in Table 3. Demarcated tumors were detected histologically in all of the cases of primary hepatic DLBCL and MALT lymphoma (Fig. 3), and no intrasinusoidal tumor invasion was found. A few atrophic reactive lymph follicles were detected in the tumors of all 20 cases. The lymphoma cells in the 12 primary hepatic DLBCL cases were positive for the following antigens (number of positive cases, % of total): CD10 (4, 33.3%), Bcl2 (6, 50%), Bcl6 (5, 41.7%), MUM1 (5, 41.7%), and CD25 (5, 41.7%) (Fig. 4a–c). Six cases had a GC B-cell phenotype. Three cases were CD5-positive and Bcl1-negative. In situ hybridization indicated nuclear EBER signals in two primary hepatic DLBCL cases (16.7%) which were complicated by rheumatic arthritis (Fig. 4d).

**Table 3:** Immunohistological findings and Epstein–Barr virus infection in 42 cases of liver-involving B-cell lymphoma

Histological type	CD5	CD10	Bcl2	Bcl6	MUM1	CD25	EBERs
Primary hepatic B-cell lymphoma							
DLBCL: 12	3 (25%)	4 (33.3%)	6 (50%)	5 (41.7%)	5 (41.7%)	5 (41.7%)	2 (16.7%)
MALT: 8	0/7 (0)	0 (0)	8 (100%)	0/6 (0)	0 (0)	0 (0)	0 (0)
Liver-involving systemic B-cell lymphoma							
IVLBCL: 12	3/10 (30%)	0/11 (0)	8/10 (80%)	1/10 (10%)	10 (83.3%)	2 (16.7%)	0 (0)
DLBCL: 10	1/9 (11.1%)	1 (10%)	8/9 (88.9%)	2/9 (22.2%)	8 (80%)	3/9 (33.3%)	0 (0)

/: positive/examined cases. *EBERs* Epstein–Barr encoded RNAs, *DLBCL* diffuse large B-cell lymphoma, *MALT* mucosa-associated lymphoid tissue, *IVLBCL* intravascular large B-cell lymphoma

The lymphoma cells of all eight MALT lymphoma cases were positive for Bcl2 and negative for CD5, CD23, CD10, Bcl6, MUM1, and CD25. No positive reaction to HBs antigen (ZCH16) was detected in the lymphoma cells of the three primary lymphoma cases who were HBs-antigen seropositive. No increase in IgG4-positive plasma cells was detected in any cases of hepatic DLBCL, MALT lymphoma, or reactive lymphoid hyperplasia.

Of the systemic B-cell lymphoma cases, all 12 IVLBCL cases had an intrasinusoidal growth pattern of lymphoma cells, and ten cases (83.3%) had MUM1-positive lymphoma cells. Eight of ten systemic DLBCL cases (80%) were positive for MUM1 and three cases (33.3%) were positive for CD25.

### Findings of 36 previously reported of primary hepatic B-cell lymphoma cases

Sixteen of the previously reported cases were DLBCL and 20 cases were MALT lymphoma [17–42]. Of the 16 DLBCL cases, nine (56.3%) were HCV-seropositive and four (25%) were HBV-seropositive. Only one DLBCL case had both HCV infection and SS. The median survival time of the ten DLBCL cases examined was 48 months. Nine of the 20 MALT lymphoma cases (45%) were HCV-seropositive and two cases (10%) were HBV-seropositive. Three MALT lymphoma cases had PBC, one had SS, and one had both PBC and SS, and these five cases (25%) did not have HCV nor HBV infection. The median survival time of the 18 MALT lymphoma cases for which this information was available was 65 months, which was significantly longer than in the above-reported ten DLBCL cases (*p* < 0.05).

## Discussion

The prevalence of HCV infection was significantly higher in the 12 primary hepatic DLBCL cases examined (66.7%) than in the systemic lymphoma cases with liver involvement, as well as in the other two control groups (*p* < 0.01). Two MALT lymphoma cases examined (25%) had chronic HCV-positive hepatitis. Bronowicki et al. [43] reported HCV infection in four of 21 primary hepatic DLBCL cases (19%), but no HCV infection in three MALT lymphoma cases. Among the previously reported cases [17–42], nine of 16 primary hepatic DLBCL cases (56.3%) and nine of 20 MALT lymphoma cases (45%) had HCV infection. Although the prevalence of HCV infection was low in the MALT lymphoma cases that we examined, our results are consistent with those of other reports [11, 43] examining the relationship between HCV infection and primary hepatic DLBCL. Intracytoplasmic HCV RNA signals have been detected in hepatocytes, mononuclear cells, and lymph follicles in the liver of HCV-seropositive cases by PCR and in situ hybridization [44]. Sung et al. [45] established a cell line of an HCV-seropositive B-cell lymphoma, from which they extracted HCV viral particles with which they were able to infect other B-cell lines as well as hepatocytes. Kasama et al. [8] demonstrated expression of the full HCV genome in B cells of HCV transgenic mice, and 25% of these mice progressed to DLBCL within 600 days after birth. These reports support the theory that HCV can infect reactive and neoplastic B cells, and that persistent HCV infection may induce primary hepatic as well as other organ B-cell lymphoma.

Of the eight HCV-seropositive primary hepatic DLBCL cases examined, five were classified as CD10- and/or Bcl6-positive GC B-cell phenotype. Expression of Bcl2 and CD25 (IL2αR) was detected in the lymphoma cells of four of these eight DLBCL cases. HCV infection has been reported to cause an approximately 5- to 10-fold increase in the mutation frequency of the Bcl6 gene in peripheral blood mononuclear cells, B-cell lymphoma cells, and their cell lines [46]. Zuckerman et al. [47] reported a significantly higher incidence of Bcl2 gene translocation in peripheral blood cells in cases with HCV-seropositive hepatitis, compared with HCV-negative controls (*p* < 0.01). Machida et al. [48] demonstrated that disruption of interferon regulatory factor 1 in HCV-infected mice increased serum IL-2 and Bcl2 expression in lymphocytes, and induced a high incidence of B-cell lymphoma. As well as inducing anti-apoptotic Bcl2 and transcription factors such as Bcl6, IL-2 may activate cytokine signaling via CD25 (IL2αR) in HCV-infected B cells [49]. Thus, lymphomagenesis may occur via a multistep mechanism in HCV-infected GC and non-GC B cells, even in the liver.

We found that two cases of primary hepatic DLBCL (16.7%) had rheumatoid arthritis treated with methotrexate. Although there were no reports of complications in these two cases, they were consistent with iatrogenic immunodeficiency-associated lymphoproliferative disorder [13]. Four of the eight cases of MALT lymphoma (50%) were complicated by PBC and/or SS or autoimmune hepatitis. Of these six cases with autoimmune disease, one case each of DLBCL and MALT lymphoma had chronic HCV hepatitis. Of the 36 previously reported cases examined [17–42], one primary hepatic DLBCL case and five MALT lymphoma cases (total 16.7%) were complicated by PBC and/or SS. Of these, only one DLBCL case had HCV infection. In Europe, SS occurs frequently in HCV-seropositive patients with type II cryoglobulinemia, which is highly suggestive of a prodromal stage of extranodal low-grade B-cell lymphoma [50, 51]. There is great geographic heterogeneity in the prevalence of type II cryoglobulinemia in HCV-seropositive cases, with type II cryoglobulinemia occurring more commonly in Southern Europe (40–60% of HCV-seropositive cases) [52]. In Japan, type III cryoglobulinemia was found in 23 of 65 HCV-seropositive cases (35.4%), but type II cryoglobulinemia was found in only one case (1.5%) [53]. Japanese lymphoplasmacytic and MALT lymphoma cases also had a low incidence of HCV infection compared with DLBCL cases [16]. These findings support the view that primary hepatic lymphoma, especially MALT lymphoma, can occur in cases of autoimmune disease independently of HCV infection and type II cryoglobulinemia in Japan.

The present study also demonstrated that HBV or EBV infection, which are important etiological factors for tumorigenesis, were a minor complication of primary hepatic B-cell lymphoma cases. Hepatic reactive lymphoid hyperplasia and IgG4-positive plasma cells have also been reported to have no direct relationship with HCV infection, autoimmune disease, or primary hepatic B-cell lymphoma [15, 54]. Although HCV infection and autoimmune disease were frequent in Japanese cases of primary hepatic B-cell lymphoma, they were mostly found independently of each other. However, primary hepatic lymphoma is rare, even in the primarily HCV-infected liver [11, 43]. Marcucci et al. [55] proposed multifactorial models for lymphomagenesis in HCV-seropositive cases, suggesting that lymphomagenesis was caused by both HCV infection and additional factors such as other receptors (CD81 and others), EBV co-infection, and environmental factors. The reasons for lymphoma being rare in the liver may be related to localized lymphoid reactions in the portal area and to specific cytotoxic immune reactions of pit (NK) cells in HCV infection [56]. Further studies are necessary to clarify the reasons for the low incidence of primary hepatic lymphoma and the mechanisms of lymphomagenesis in HCV infection.

The 5-year survival rate of the nine primary hepatic DLBCL cases we examined was 43%, and the five cases receiving rituximab plus cytotoxic treatment were alive and well at the time of writing. A French lymphoma study demonstrated that 24 HCV-seropositive systemic DLBCL cases had a significantly worse overall 2-year survival rate (56%) than 72 HCV-negative DLBCL cases (80%), after matching for age and prognostic factors (*p* = 0.02) [57]. However, four reported primary hepatic DLBCL cases had a good prognosis after cytotoxic treatment with or without rituximab [58]. Antiviral treatment after cytotoxic treatment also contributed to a significantly longer disease-free survival period in 69 HCV-seropositive B-cell lymphoma cases (*p* < 0.05) [59]. These data strongly suggest that rituximab plus cytotoxic treatment followed by antiviral treatment may result in an improved clinical outcome in primary hepatic and systemic HCV-seropositive DLBCL cases.
